# Supplementary figures and images for: Comprehensive mutagenesis to identify amino acid residues contributing to the difference in thermostability between two originally thermostable ancestral proteins
Source: PLoS One. 2021 Oct 21;16(10):e0258821. doi: 10.1371/journal.pone.0258821 (PMC8530338; doi:10.1371/journal.pone.0258821)

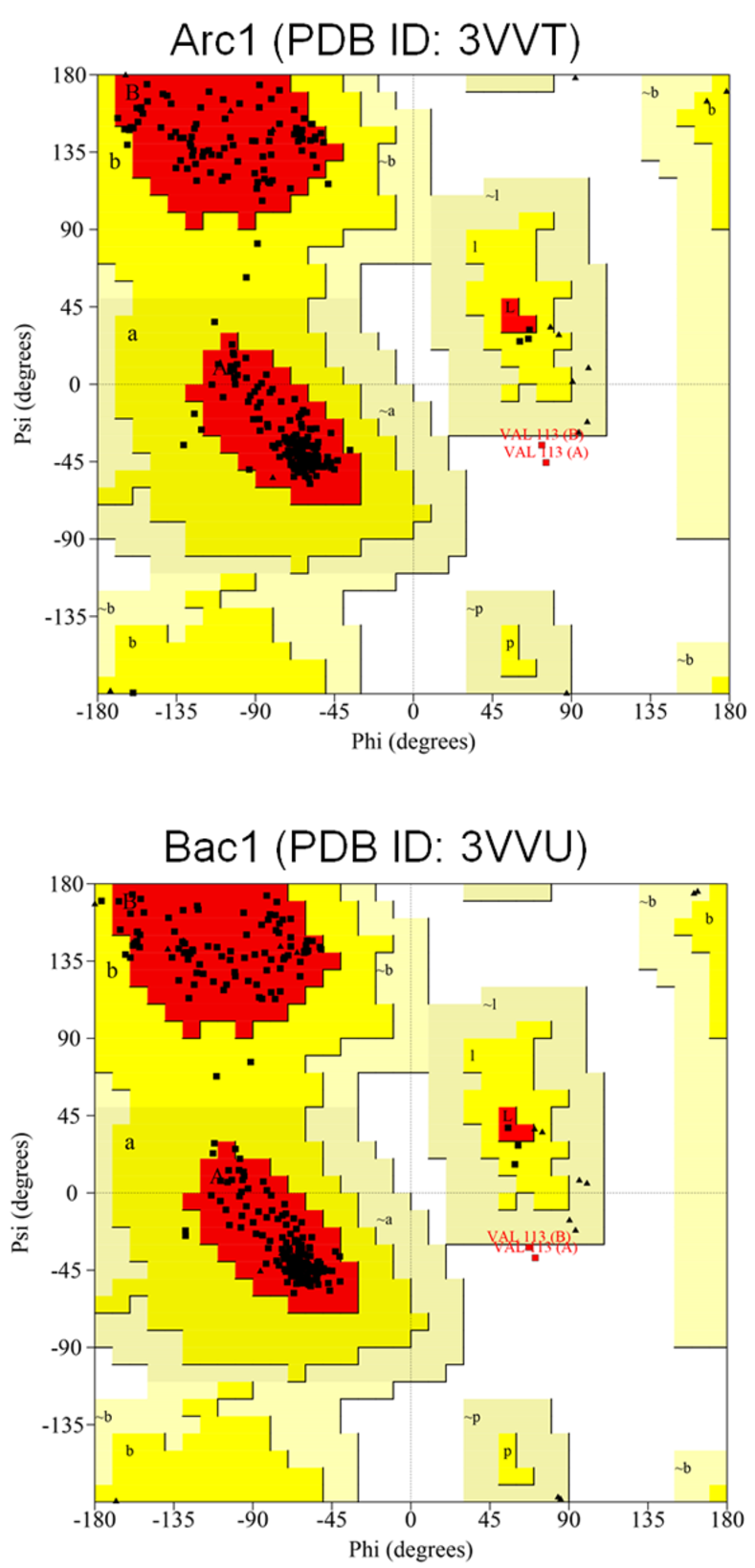

Supplement: S1 Fig — The most favored regions are represented by red; additional allowed regions are represented by yellow; generously allowed regions are represented by light yellow; disallowed regions are represented by white. Glycine residues are shown as triangles and the other residues are shown as squares. In each plot, only Val113 exists in disallowed regions. (TIF) [file pone.0258821.s001.tif]

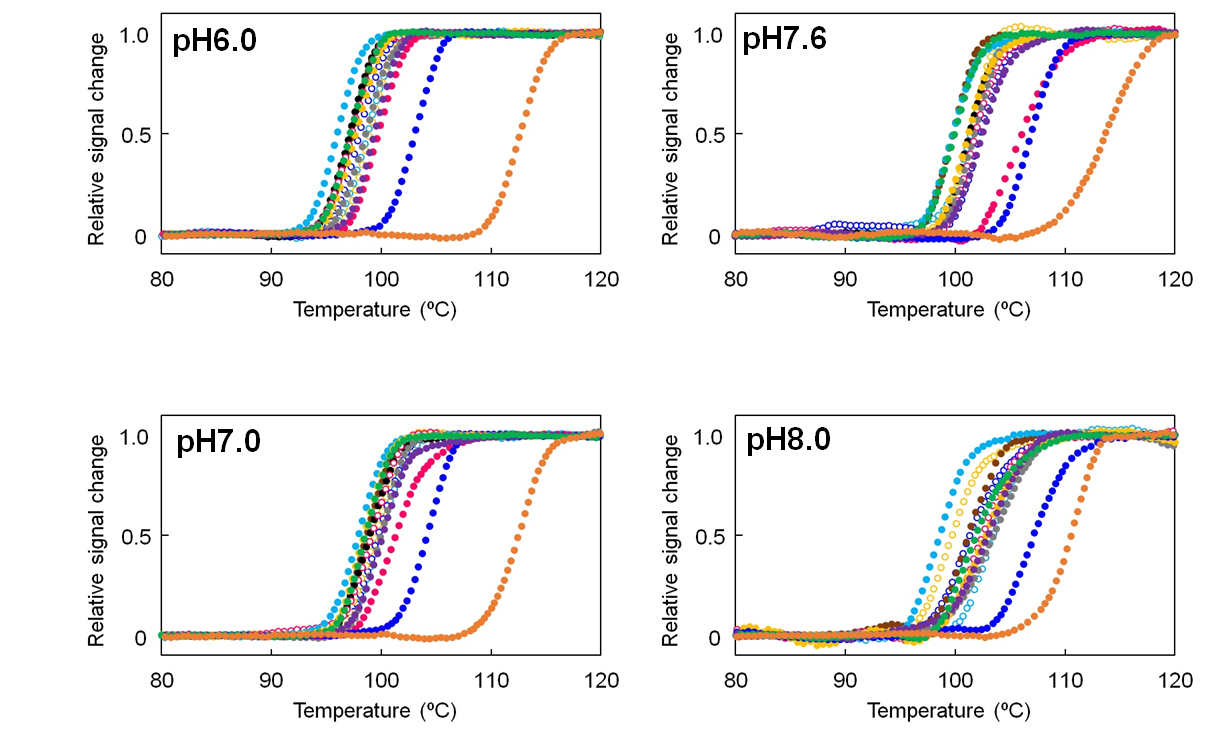

Supplement: S2 Fig — The change in ellipticity at 222 nm was monitored as a function of temperature. The scan rate was 1.0°C/min. The samples comprised 20 μM protein in 20 mM potassium phosphate, 50 mM KCl, 0.5 mM EDTA. The plots were normalized with respect to the baseline of the native and unfolded states. Orange filled circles, Arc1; green filled circles, Bac1; cyan open circles, F30L; blue open circles, L37M; yellow open circles, Q42R; brown filled circles, L44M; black filled circles, G60A; magenta open circles, F64Y; gray filled circles, V80A; cyan filled circles, I88V; yellow filled circles, M107L; magenta filled circles, S108D; blue, G116A; purple filled circles, L120P. (TIF) [file pone.0258821.s002.tif]

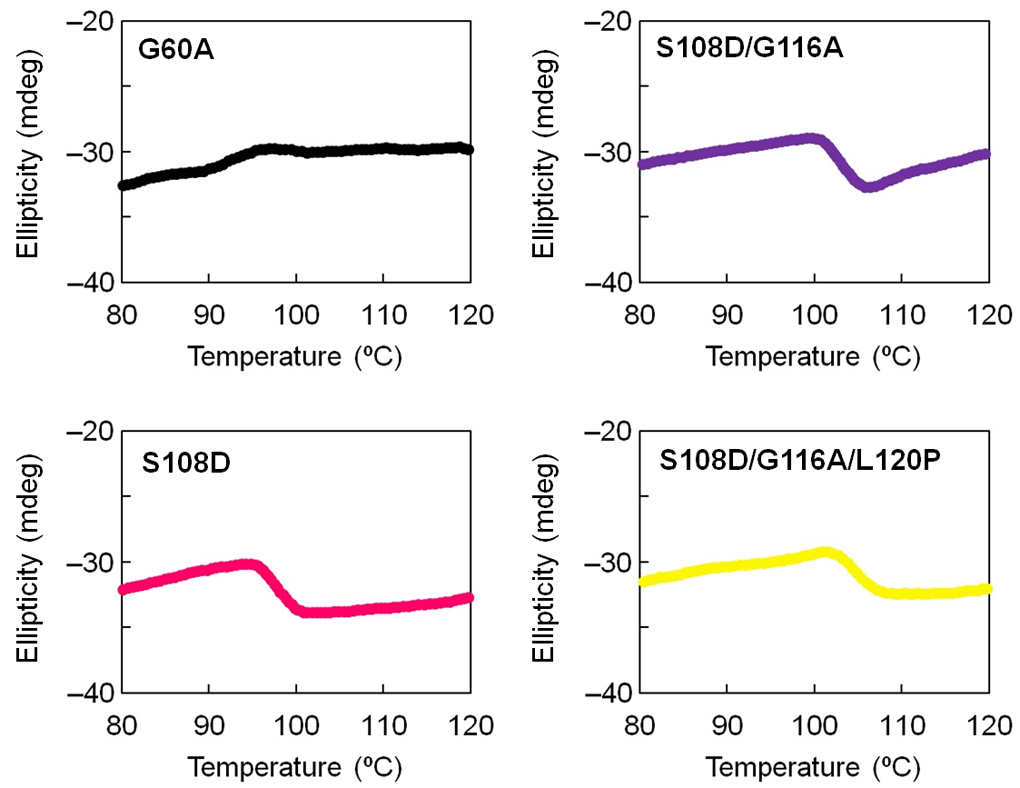

Supplement: S3 Fig — The raw data for the change in ellipticity at 222 nm are presented as a function of temperature. The scan rate was 1.0°C/min. The samples comprised 20 μM protein in 20 mM potassium phosphate (pH 8.0), 50 mM KCl, 0.5 mM EDTA. (TIF) [file pone.0258821.s003.tif]

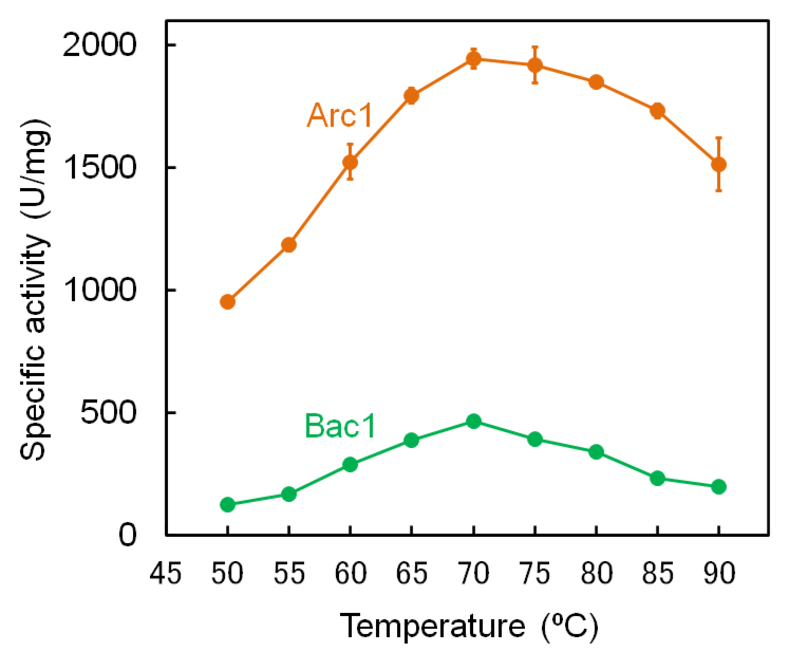

Supplement: S4 Fig — The specific activities were measured using a reaction mixture consisting of 50 mM HEPES (pH 8.0), 25 mM KCl, 10 mM (NH4)2SO4, 2.0 mM Mg(CH3COO)2, 1.0 mM dithiothreitol, 1.0 mM ADP and 2.5 mM GTP. One enzyme unit equaled 1 μmol ATP formed per min. Each value is the average of at least three replicas. (TIF) [file pone.0258821.s004.tif]

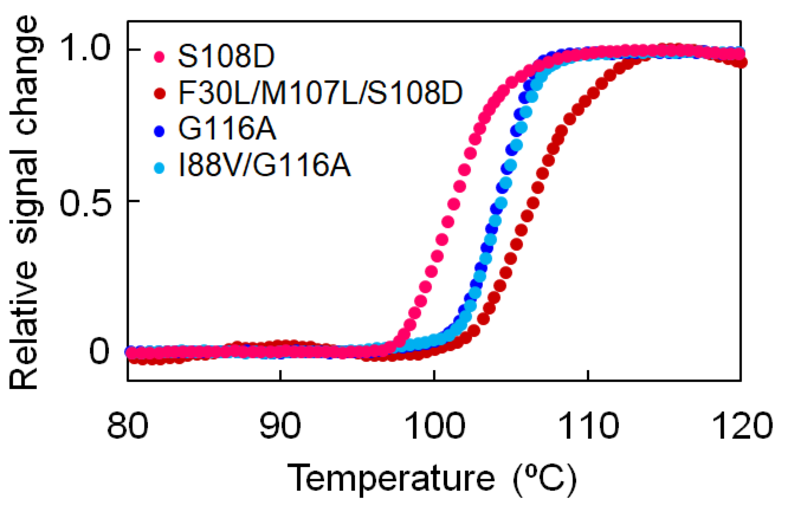

Supplement: S5 Fig — The change in ellipticity at 222 nm was monitored as a function of temperature. The scan rate was 1.0°C/min. The samples comprised 20 μM protein in 20 mM potassium phosphate (pH 7.0), 50 mM KCl, 0.5 mM EDTA. The plots were normalized with respect to the baseline of the native and denatured states. Magenta, S108D; red, F30L/M107L/S108D; blue, G116A; cyan, I88V/G116A. (TIF) [file pone.0258821.s005.tif]

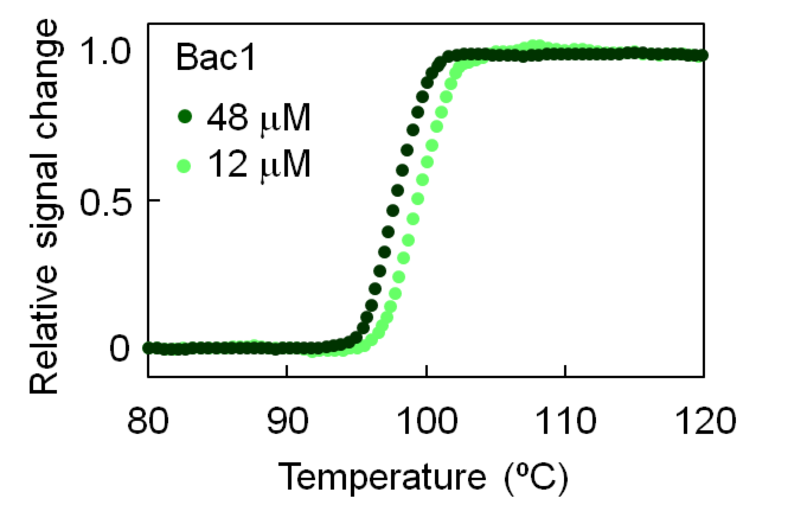

Supplement: S6 Fig — Bac1 was dissolved in 20 mM potassium phosphate, pH 7.0, 50 mM KCl, 1 mM EDTA. The protein concentration was 12 μM (light green) or 48 μM (dark green). The scan rate was 1.0°C/min. The plots were normalized with respect to the baseline of the native and denatured states. (TIF) [file pone.0258821.s006.tif]

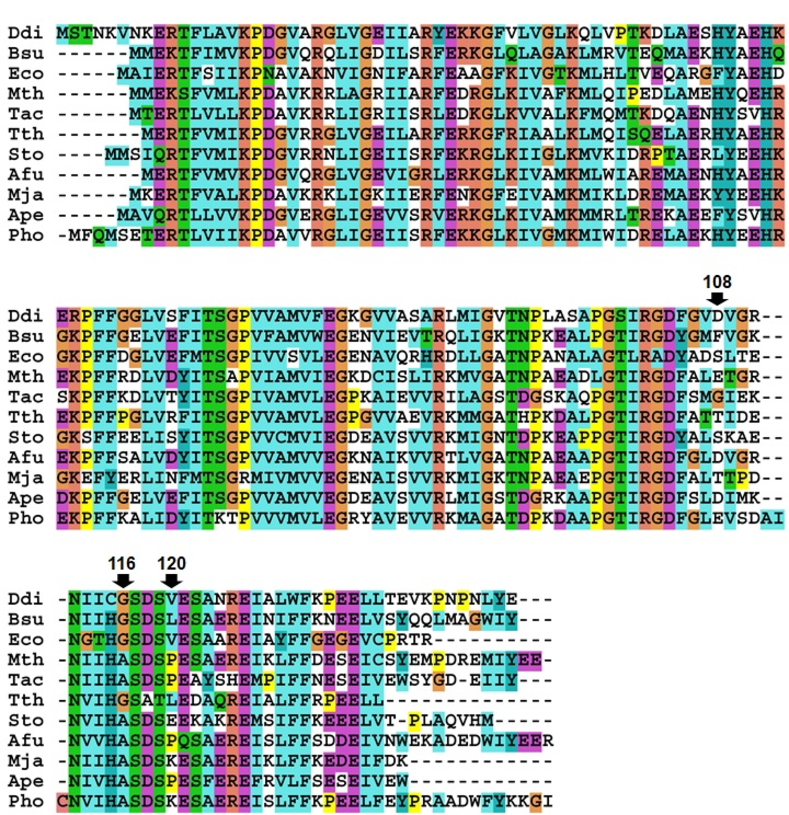

Supplement: S7 Fig — Positions corresponding to those at 108, 116 and 120 of the ancestral NDKs are indicated above the sequences. The sequences were aligned with MAFFT (https://mafft.cbrc.jp/alignment/server/) and visualized using ClustalX (http://www.clustal.org/clustal2/). (TIF) [file pone.0258821.s007.tif]
