# Supplementary material for: Comprehensive mutagenesis to identify amino acid residues contributing to the difference in thermostability between two originally thermostable ancestral proteins
Source: PLoS One. 2021 Oct 21;16(10):e0258821. doi: 10.1371/journal.pone.0258821 (PMC8530338; doi:10.1371/journal.pone.0258821)
Supplement: S1 Table — (DOCX) [file pone.0258821.s008.docx]

**S1 Table.** Mutagenic primers used in this study.

| Primer | Sequence (5’→3’) ^a^ |
| --- | --- |
| F30L_forward | CGTTTCGAACGTAAAGGTCTGAAAATCGTTGCTATGAAAC |
| F30L_reverse | GTTTCATAGCAACGATTTTCAGACCTTTACGTTCGAAACG |
| L37M_forward | CAAAATCGTTGCTATGAAAATGATGCGTATCTCTCAGGA |
| L37M_ reverse | TCCTGAGAGATACGCATCATTTTCATAGCAACGATTTTG |
| Q42R_forward | CTGATGCGTATCTCTCGTGAACTGGCTGAAAAACAC |
| Q42R_ reverse | GTGTTTTTCAGCCAGTTCACGAGAGATACGCATCAG |
| L44M_forward | TGCGTATCTCTCAGGAAATGGCTGAAAAACACTACGC |
| L44M_ reverse | GCGTAGTGTTTTTCAGCCATTTCCTGAGAGATACGCA |
| G60A_forward | GAAAAACCGTTCTTCTCTGCTCTGGTTGACTTCATCAC |
| G60A_ reverse | GTGATGAAGTCAACCAGAGCAGAGAAGAACGGTTTTTC |
| F64Y_forward | CTCTGGTCTGGTTGACTACATCACCTCTGGTCCGG |
| F64Y_ reverse | CCGGACCAGAGGTGATGTAGTCAACCAGACCAGAG |
| V80A_forward | GTTCTGGAAGGTAAAAACGCTGTTGAAGTTGTTCGTAAAA |
| V80A_ reverse | TTTTACGAACAACTTCAACAGCGTTTTTACCTTCCAGAAC |
| I88V_forward | TGAAGTTGTTCGTAAAATGGTTGGTGCTACCAACCCG |
| I88V_ reverse | CGGGTTGGTAGCACCAACCATTTTACGAACAACTTCA |
| M107L_forward | CCGTGGTGACTTCGGTCTGTCTGTTGGTAAAAACGTTA |
| M107L_ reverse | TAACGTTTTTACCAACAGACAGACCGAAGTCACCACGG |
| S108D_forward | GTGGTGACTTCGGTATGGACGTTGGTAAAAACGTTATCC |
| S108D_ reverse | GGATAACGTTTTTACCAACGTCCATACCGAAGTCACCAC |
| G116A_forward | GGTAAAAACGTTATCCACGCTTCTGACTCTCTGGAATC |
| G116A_ reverse | GATTCCAGAGAGTCAGAAGCGTGGATAACGTTTTTACC |
| L120P_forward | CCACGGTTCTGACTCTCCGGAATCTGCTGAACGTGA |
| L120P_ reverse | TCACGTTCAGCAGATTCCGGAGAGTCAGAACCGTGG |
| L120P(G116A)_forward | CCACGCTTCTGACTCTCCGGAATCTGCTGAACGTGA |
| L120P(G116A)_ reverse | TCACGTTCAGCAGATTCCGGAGAGTCAGAAGCGTGG |
| M107L/S108D_forward | CCGTGGTGACTTCGGTCTGGACGTTGGTAAAAACGTTATCC |
| M107L/S108D_ reverse | GGATAACGTTTTTACCAACGTCCAGACCGAAGTCACCACGG |

^a^ The mutated sequences are underlined. L120P(G116A)_forward and L120P(G116A)_reverse were used to synthesize the gene for S108D/G116A/L120P with pET21c harboring the gene for S108D/G116A as the template.
