# Supplementary material for: Comprehensive mutagenesis to identify amino acid residues contributing to the difference in thermostability between two originally thermostable ancestral proteins
Source: PLoS One. 2021 Oct 21;16(10):e0258821. doi: 10.1371/journal.pone.0258821 (PMC8530338; doi:10.1371/journal.pone.0258821)
Supplement: S2 Table — (DOCX) [file pone.0258821.s009.docx]

**S2 Table.** The numerical data for *T*_m_, *K*_m_, *k*_cat_ and *k*_cat_/*K*_m_ of Arc1, Bac1 and its mutants.

|  | *T*_m_ (ºC)^a^ | | | | *K*_m_^b^  (μM) | *k*_cat_^b^  (s^-1^) | *k*_cat_/*K*_m_  (s^-1^ μM^-1^) |
| --- | --- | --- | --- | --- | --- | --- | --- |
|  | pH6.0 | pH7.0 | pH7.6 | pH8.0 |  |  |  |
| Arc1 | 113 | 113 | 114 | 111 | 430 ± 70 | 1800 ± 100 | 4.1 |
| Bac1 | 97 | 99 | 100 | 102 | 180 ± 40 | 370 ± 30 | 2.0 |
| F30L | 99 | 100 | 101 | 103 | 350 ± 30 | 740 ± 30 | 2.1 |
| L37M | 98 | 100 | 103 | 101 | 300 ± 70 | 570 ± 50 | 1.9 |
| Q42R | 99 | 100 | 101 | 100 | 240 ± 10 | 310 ± 0 | 1.3 |
| L44M | 97 | 99 | 100 | 101 | 270 ± 40 | 320 ± 20 | 1.2 |
| G60A | 97 | 99 | 101 | – ^c^ | 200 ± 20 | 640 ± 20 | 3.2 |
| F64Y | 97 | 99 | 102 | 103 | 360 ± 40 | 1000 ± 100 | 2.9 |
| V80A | 99 | 100 | 102 | 103 | 140 ± 10 | 380 ± 0 | 2.6 |
| I88V | 96 | 98 | 100 | 99 | 190 ± 40 | 400 ± 30 | 2.1 |
| M107L | 97 | 98 | 101 | 103 | 160 ± 30 | 220 ± 10 | 1.4 |
| S108D | 100 | 102 | 106 | – ^c^ | 180 ± 10 | 420 ± 10 | 2.3 |
| G116A | 103 | 104 | 107 | 107 | 260 ± 40 | 460 ± 30 | 1.8 |
| L120P | 100 | 101 | 103 | 103 | 160 ± 20 | 250 ± 10 | 1.5 |
| S108D/G116A | 105 | 107 | 112 | – ^c^ | 310 ± 30 | 460 ± 20 | 1.5 |
| S108D/G116A/L120P | 108 | 110 | 113 | – ^c^ | 250 ± 50 | 420 ± 30 | 1.7 |
| Bac1mu9 | 103 | 105 | 107 | 106 | 500 ± 30 | 720 ± 20 | 1.4 |

^a^ The *T*_m_ values were estimated from the data shown in S2 Fig and Fig 4.

^b^ *K*_m_ for ADP, *k*_cat_, and standard errors were calculated by nonlinear least-square fitting of the steady-state kinetic data to the Michaelis-Menten equation using the Enzyme Kinetics module of SigmaPlot Ver. 13 (Systat Software, Richmond).

^c^ *T*_m_ values are not presented for G60A, S108D, S108D/G116A and S108D/G116A/L120P at pH 8.0 because atypical unfolding curves were observed for the proteins at pH 8.0 (S3 Fig).
